# Microsatellite instability in colorectal cancer

**DOI:** 10.17179/excli2017-948

**Published:** 2018-01-22

**Authors:** Jafar Nouri Nojadeh, Shahin Behrouz Sharif, Ebrahim Sakhinia

**Affiliations:** 1 Department of Medical Genetics, Faculty of Medicine, Tabriz University of Medical Sciences, Tabriz, Iran; 2 Stem Cell and Regenerative Medicine Institute, Tabriz University of Medical Sciences, Tabriz, Iran; 3 Department of Molecular Medicine, Pasteur Institute of Iran, Tehran; 4 Tuberculosis and Lung Disease Research Center, Tabriz University of Medical Sciences, Tabriz, Iran; 5 Tabriz Genetic Analysis Centre (TGAC), Tabriz University of Medical Sciences, Tabriz, Iran

**Keywords:** CRC, MSI, DNA MMR system

## Abstract

Colorectal cancer (CRC) is a heterogeneous disease that is caused by the interaction of genetic and environmental factors. Although it is one of the most common cancers worldwide, CRC would be one of the most curable cancers if it is detected in the early stages. Molecular changes that occur in colorectal cancer may be categorized into three main groups: 1) Chromosomal Instability (CIN), 2) Microsatellite Instability (MSI), and 3) CpG Island Methylator phenotype (CIMP). Microsatellites, also known as Short Tandem Repeats (STRs) are small (1-6 base pairs) repeating stretches of DNA scattered throughout the entire genome and account for approximately 3 % of the human genome. Due to their repeated structure, microsatellites are prone to high mutation rate. Microsatellite instability (MSI) is a unique molecular alteration and hyper-mutable phenotype, which is the result of a defective DNA mismatch repair (MMR) system, and can be defined as the presence of alternate sized repetitive DNA sequences which are not present in the corresponding germ line DNA. The presence of MSI is found in sporadic colon, gastric, sporadic endometrial and the majority of other cancers. Approximately, 15-20 % of colorectal cancers display MSI. Determination of MSI status in CRC has prognostic and therapeutic implications. As well, detecting MSI is used diagnostically for tumor detection and classification. For these reasons, microsatellite instability analysis is becoming more and more important in colorectal cancer patients. The objective of this review is to provide the comprehensive summary of the update knowledge of colorectal cancer classification and diagnostic features of microsatellite instability.

## Introduction

Colorectal cancer (CRC) is the third most prevalent cancer in humans and the third most common cause of cancer related deaths in both males and females that contributes to a significant public health problem worldwide (Siegel et al., 2012[45]; Jemal et al., 2011[27]). The majority of colorectal cancer cases are sporadic (about 75 %) that display no apparent evidence of having inherited disorders, suggesting contribution of genetic and environmental factors, whereas only 25 % of the patients have family histories of the disease (Jasperson et al., 2010[26]). Only 5-6 % of patients with colorectal cancer with a family background are due to inherited mutations in major CRC genes, while the rest are the result of accumulation of both genetic mutations and epigenetic modifications of several genes (Migliore et al., 2011[35]). According to the current knowledge, CRC mainly develops through a gradual accumulation of genetic and epigenetic alterations of the genome (Fearon and Vogelstein, 1990[16]). Molecular changes that occur in colorectal cancer may be categorized into three main groups: 1) Chromosomal Instability (CIN), 2) Microsatellite Instability (MSI), and 3) CpG Island Methylator phenotype (CIMP) that silences gene function with aberrant hypermethylation (Worthley and Leggett, 2010[53]). CIN-positive CRCs are featured with alterations in the structure and number of chromosome besides increased mutation rates in both tumor suppressor genes and oncogenes. Actually, mutation rates in single nucleotides are higher in MSI-positive CRCs than in CIN-positive CRCs (Cancer Genome Atlas Network, 2012[9]). On the other hand, 15-20 % of CRCs display MSI caused by defective DNA mismatch repair (MMR) system (Vilar and Gruber, 2010[51]). In this review, we provide the comprehensive summary of the current knowledge in the field of colorectal cancer classification and diagnostic features of microsatellite instability.

## Types of CRC and their Genetic Basis

According to etiology and genetics of the disease, CRC is usually classified into three distinct groups: sporadic, familial, and hereditary (Table 1(Tab. 1)) (Sameer, 2013[40]).

### Sporadic CRC

Sporadic colorectal cancer is the most common type of CRC and includes approximately 75 % of cases that display no apparent evidence of having inheritance of disorder. However, this is unclear, since genetic factors seem to affect the likelihood of cancer even in the absence of specific mutations. Sporadic colorectal cancer is common among elder people, probably as a result of environmental factors, dietary, and aging (Arvelo et al., 2015[3]). MSI-H sporadic colorectal cancers are the most often (about 70 %-95 %) caused by alteration of MLH1 gene via somatic promotor hypermethylation (Copija et al., 2017[13]).

### Familial type of CRC

This type of CRC is often considered sporadic and not any associated gene has been identified yet. People with a history of colorectal cancer in a first-degree relative are at increased risk of two to three times higher than the normal population (Lin, 2012[31]).

### Hereditary type of CRC

The hereditary type of CRC is divided into five subtypes:

### Familial adenomatous polyposis (FAP)

Familial adenomatous polyposis is the most common hereditary polyposis syndrome, which is an autosomal dominant disease caused by a mutation in the APC gene on chromosome 5q21. The APC gene is a tumor suppressor gene that produces APC protein, a multifunction protein which controls how quickly cells grow and prevent the development of tumors. The normal function of APC protein is the regulation of β-catenin by its degradation. β-catenin plays an important role in cell communication, Wnt signalling pathway, and growth by acting as a transcription factor for proliferation genes. Mutations in the APC gene lead to loss of APC function and result in an accumulation of β-catenin. Many different mutations (e.g. insertions, deletions, nonsense mutations) of the APC gene are described as a cause of FAP (Bogaert and Prenen, 2014[4]). In addition to APC mutation, other mutations, such as K-RAS, DCC, P53, COX-2, BCL-2 and etc. are required to develop cancer (Zeichner et al., 2012[57]). In FAP patients, polyps are mainly found in the proximal colon and rarely in the rectum. The average age for people with FAP to develop polyps is 35 years and if FAP is not recognized and treated, most likely it will develop colorectal cancer (Laurent et al., 2011[29]). There are four subtypes of FAP that are caused by different germ line mutations in the APC gene: Attenuated FAP (AFAP), Gardner syndrome, Turcot syndrome, and Gastric Adenocarcinoma and Proximal Polyposis of the Stomach (GAPPS).

### MUTYH-associated polyposis (MAP)

MUTYH-associated polyposis is one of the hereditary polyposis syndromes which is an autosomal recessive disease and is caused by a biallelic germline mutation in MUTYH gene on chromosome 1p34.1. MUTYH gene encodes an enzyme called MYH glycosylase that is involved in a DNA repair system called Base Excision Repair (BER). The number of polyps in MAP disease is less than FAP and it is phenotypically similar to attenuated FAP (Goodenberger and Lindor, 2011[21]). The average age for diagnosis of MAP is between 40-60 years old and if MAP is not recognized and treated, patients will have an 80 % risk of developing CRC (Theodoratou et al., 2010[49]). The majority of studied CRCs in people with MAP were microsatellite stable; although the MSI-H (high-level microsatellite instability) phenotype is reported in the minority of CRCs of persons with MAP (Nielsen et al., 2011[36]; Castillejo et al., 2014[10]). 

### Peutz-Jeghers syndrome (PJS)

Peutz-Jeghers syndrome is a rare autosomal dominant disorder, which is one of the hereditary polyposis syndromes and is characterized by multiple benign hamartomatous polyps in the gastrointestinal tract, most often found in the small intestine. The number of polyps in PJS is less than MAP syndrome and those polyps are present from childhood (Giardiello and Trimbath, 2006[19]). The major cause of this disease is germ line mutations in the STK11 (serine threonine kinase 11) gene, also known as the LKB1, which is a tumor suppressor gene and is located on chromosome 19p13.3 (Chae and Jeon, 2014[11]). Mutations in this gene change the structure and/or function of the STK11 protein, disrupting its ability to restrain cell division with the loss of kinase activity. Microsatellite instability, LOH nearby the APC gene, and KRAS mutations have been identified in some tumors (Shah and Lindor, 2010[42]).

### Serrated polyposis syndrome (SPS)

Serrated polyposis syndrome is a relatively rare syndrome characterized by multiple serrated polyps of the colon, which was previously known as the hyperplastic polyposis syndrome (Sweetser et al., 2013[47]). This syndrome is usually considered sporadic and underlying genetic causes are related to germline mutations of oncogene-induced senescence pathway genes (Gala et al., 2014[18]). These tumors will be more often considered MSI-low or MSS (Microsatellite Stable) (Guarinos et al., 2012[22]). There are three criteria for diagnosis of SPS and an individual would be considered affected if have at least one of them; A) at least five serrated polyps proximal to sigmoid with at least two of them being greater than 1 cm; B) Any number of serrated polyps occurring proximal to the sigmoid colon in an individual who has a first-degree relative with SPS; and C) more than 20 serrated polyps distributed throughout the colon. Furthermore, it would be noteworthy to indicate that SPS has been associated with an increased risk of developing CRC considering previous studies (Rex et al., 2012[37]).

### Lynch syndrome (LS)

Lynch syndrome (LS), also was known as Hereditary non-polyposis colorectal cancer, is the most common hereditary colon cancer syndrome which is an autosomal dominant disease and is caused by germline mutations in one of several DNA mismatch repair (MMR) genes, including MSH2 on chromosome 2p16, MLH1 on chromosome 3p21, MSH6 on chromosome 2p16, and PSM2 on chromosome 7p22 (Lynch et al., 2009[32]). MSH2 and MLH1 mutations account for the majority of Lynch syndrome cases (Lynch and Shaw, 2013[33]). MMR genes encode proteins that are critical to the suitable repair of DNA sequence mismatch and correct base mismatches or small deletions or insertions (Shi and Washington, 2012[43]). Inactivation of these genes interrupts DNA repair and causes an alteration in the short-tandem DNA repetitive sequences or microsatellites, resulting in the development of a phenotype known as microsatellite instability which is a hallmark of Lynch syndrome. This syndrome is accounting for approximately 2-3 % of the total CRC cases (Hampel et al., 2005[24]). High-level microsatellite instability is observed in approximately 90 % of LS-associated CRCs (Boland and Shike, 2010[6]).

## Consensus Molecular Subtypes of CRC

To resolve inconsistencies between CRC classifications based on gene expressions reported and simplify clinical translation four Consensus Molecular Subtypes (CMSs) with distinct features have been reported. The CMS1 are (microsatellite instability immune, 14 %), hypermutated, microsatellite unstable and are immunogenic. The CMS2 are (canonical, 37 %), epithelial, marked WNT and MYC signalling activation and have the highest overall survival. The CMS3 are (metabolic, 13 %), epithelial and evident metabolic cancer phenotype; and the CMS4 (mesenchymal, 23 %), prominent transforming growth factor-β activation, stromal invasion and angiogenesis that have a worst survival. The Consensus Molecular Subtypes of CRC with clear biological interpretability may have a better prognosis, therapeutic response, and potential new treatment strategies (Guinney et al., 2015[23]; Thanki et al., 2017[48]). 

## Molecular Basis of DNA Mismatch Repair System

**Figure 1:**
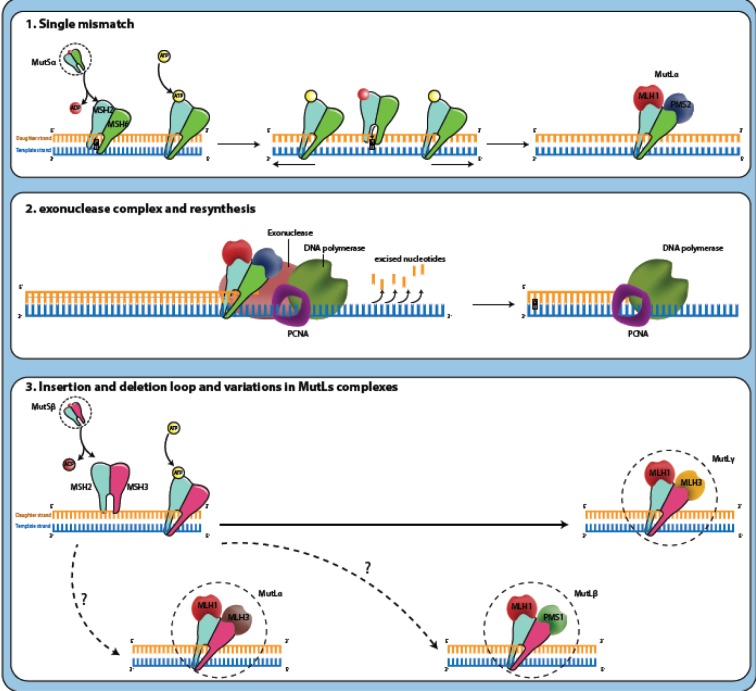
Mechanism of mismatch repair system: (1) MutSα or MutSβ has recognized the mismatched DNA base pairs during replication that the DNA polymerase has matched the mistake base G (guanosine) in daughter strand with the T (thymidine) on the template. MutSα or MutSβ can recruit MutLα, MutLβ or MutLγ heterodimers by means of exchanging ATP to ADP. This complex (MutS-MutL) creates a sliding clamp around the DNA and moves along the new DNA chain when it encounters the DNA polymerase complex. (2) The proteins in sliding clamp interact with exonuclease-1 (EXO1) and proliferating cell nuclear antigen (PCNA). This complex excises the daughter strand back to the site of the mismatch. Finally, re-synthesize and re-ligation are performed by DNA polymerase and DNA ligase, respectively. The correction occurs (Boland and Goel, 2010).

DNA mismatch repair system (MMR) corrects erroneous insertion, deletion, and base-base mismatches generated during DNA replication and recombination that have escaped the proofreading process (Jiricny, 2006[28]). This repair pathway is highly conserved from bacteria to humans and safeguards the integrity of the genome (Hsieh and Yamane, 2008[25]). MutS and MutL are the main proteins involved in prokaryote MMR system that function as homodimers whereas, in eukaryotes, MSH2, MSH3, and MSH6 are homologs for MutS; MLH1, MLH2, MLH3 are MutL homologs. There are also other homologs for MutL (post-meiotic segregation) named PMS1 and PMS2 which interact as heterodimers (Fukui, 2010[17]). When a mismatch is detected in the eukaryotic genome, DNA mismatch repair system functions through a series of steps: MSH2 associates with MSH6 or MSH3 causing the formation of MutSα and MutSβ heterodimers, respectively. MutSα recognizes single base mismatches and small insertion/deletion loops (IDLs), while MutSβ recognizes larger loops. MutSα or MutSβ can recruit MutLα, MutLβ or MutLγ heterodimers (if MLH1 couples with PMS2, PMS1 or MLH3, respectively) by means of exchanging adenosine triphosphate (ATP) to adenosine diphosphate (ADP). This complex (MutS-MutL) creates a sliding clamp around the DNA. The proteins in sliding clamp interact with exonuclease-1 and proliferating cell nuclear antigen (PCNA). This complex excises the daughter strand back to the site of the mismatch. Finally, re-synthesize and re-ligation are performed by DNA polymerase and DNA ligase, respectively. The correction occurs (Figure 1(Fig. 1), Reference in Figure 1: Boland and Goel, 2010[5]) (Li, 2008[30]; Martín-López and Fishel, 2013[34]).

## Microsatellite Instability (MSI)

Microsatellites, also known as Short Tandem Repeats (STRs) are small (1-6 base pairs) repeating stretches of DNA scattered throughout the entire genome (both in coding and non-coding regions) and account for approximately 3 % of the human genome. Due to their repeated structure, microsatellites are prone to high mutation rate (Ellegren, 2004[15]). Microsatellite instability in tumor DNA is defined as the presence of alternate sized repetitive DNA sequences that are not present in the corresponding germline DNA. Microsatellite instability (MSI) is a molecular phenotype due to a defective DNA mismatch repair system. 

The presence of MSI is found in the sporadic colon, gastric, sporadic endometrial and the majority of other cancers (Yamamoto and Imai, 2015[55]). Determination of MSI status in CRC has prognostic and therapeutic implications. As well, MSI can be used diagnostically for tumor detection and classification (Setaffy and Langner, 2015[41]). MSI has always been associated with an improved prognosis, stage for stage. Recently the reason for this has been discovered, a reason that has changed our approach to advanced MSI-high disease. The unstable microsatellites are highly immunogenic, so that therapy that activates the immune system can have almost miraculous effects on unstable tumors. This has led to proposals to develop tumor vaccines and to turn MSS tumors into MSI to make them more immunogenic.

## Detection of MSI

MSI is detected indirectly by analysis of MMR protein expression by Immunohistochemical (IHC) staining, or directly by PCR-based amplification of specific microsatellite repeats, which is the most common method to detect MSI (Buecher et al., 2013[7]). 

### IHC method

Immunohistochemical analysis can determine loss of expression of one or more of MMR proteins. Actually, IHC is correlates with MSI but it is not a perfect test for MSI determination. It is a test of expression of mismatch repair proteins in cells. In this method, antibodies against MMR proteins such as MLH1, MSH2, PMS2 and MSH6 provide information of the MMR system functionality. IHC analysis with PMS2 and MSH6 antibodies is able to detect most abnormalities in the corresponding encoding genes as well as mutations in MLH1 and MSH2; however, IHC assay with MLH1/MSH2 antibodies can detect a fraction of MLH1 or MSH2 abnormalities but not all of them. Therefore, IHC analysis with MSH6 and PMS2 antibodies has more diagnostic potential than analysis with MLH1 and MSH2 antibodies (Shia, 2008[44]). The main relevance of MSI and IHC is as a screening test for Lynch Syndrome. Universal MSI/IHC on tumors is increasingly performed throughout the world.

### PCR-based method

For MSI analysis by the fluorescent multiplex PCR-based method, we need DNA from tumor tissues and normal tissues, a series of primers one of which is fluorescently end labeled (the sense strand or antisense strand of each primer), a sequencer, and appropriate software. The principle of this method is to measure the presence of different lengths of specific microsatellite markers in tumor cells comparing to normal cells (Setaffy and Langner, 2015[41]). 

In the first attempt to the diagnosis of MSI in CRC, a consensus conference recommended a panel of microsatellite markers included three dinucleotide repeats (D5S346, D2S123, and D17S250) and 2 mononucleotide repeats (BAT25 and BAT26). Three distinct MSI phenotypes have been described. If two or more microsatellite markers are mutated, the tumor is considered MSI-high (MSI-H); if only one is mutated, the tumor is defined as MSI-low (MSI-L); and if none of the examined loci demonstrate instability, the tumor will be considered Microsatellite Stable (MSS). This panel was known as the Bethesda panel (Rodriguez-Bigas et al., 1997[38]).

A few years later, it was found that mononucleotide markers have a better specificity and sensitivity than dinucleotide repeats (dinucleotide markers have a polymorphic nature) (Suraweera et al., 2002[46]) and hence Bethesda guideline criteria were revised by NCI (National Cancer Institute) at the following conference in 2004 (Umar et al., 2004[50]). After that, the uses of panels containing more mononucleotide markers have been increased due to their higher sensitivity and specificity in the diagnosis of MSI in CRCs (Table 2(Tab. 2)) (Buhard et al., 2004[8]; Xicola et al., 2007[54]; Goel et al., 2010[20]; You et al., 2010[56]; Agostini et al., 2010[2]; Cicek et al., 2011[12]).

## MSI in Treatment

The uses of MSI status in the prediction of response to adjuvant chemotherapy is controversial although it has been confirmed that colorectal tumors displaying MSI have a better prognosis compared with MSS tumors. Antimetabolites (5-flourouracil), Alkylating agents and Topoisomerase Inhibitors are the three categories of chemotherapeutic agents that are used in CRC treatment (Warusavitarne and Schnitzler, 2007[52]). The chemotherapeutic treatment is effective in some certain patients, but it can cause many adverse effects, nonetheless (Rothenberg et al., 2001[39]; Adlard et al., 2002[1]). MSI-H is one of the potential predictive points to the chemotherapeutic treatment efficacy and to the level of adverse effects in a patient; therefore, several clinical trials have been conducted regarding this opinion (De la Chapelle and Hampel, 2010[14]). There are different therapeutic responses in MSI-H CRCs depending on type of adjuvant chemotherapy. When a tumor is to be MSI-high, for diagnosis it is either Lynch or Methylated. If IHC is done and the unexpressed protein is MSH2, PMS2 or MSH6 then it is Lynch. Germline testing is indicated. If the unexpressed protein is MLH1 it could be a CIMP tumor with hypermethylation of MLH1 promoter, or Lynch. To tell which is which, BRAF mutation testing or methylation assay on the tumor are helpful. For treatment it is a candidate for immune activation therapy if it is advanced. If not advanced it has a good prognosis and will not respond to 5 FU based therapy.

## Conclusion and Concluding Remark

Since CRC is one of the most prevalent cancers in humans and causes a remarkable public health problem worldwide, identifying the ways of diagnosis and treatment of CRC is of most importance. MSI is a significant genetic marker in CRC that can be useful in diagnosis, prognosis, and prediction of chemotherapeutic treatment efficacy. Nowadays, molecular techniques have been developed for detection of MSI and drug development strategies are focused on specific tumor molecular characteristics.

## Figures and Tables

**Table 1:**
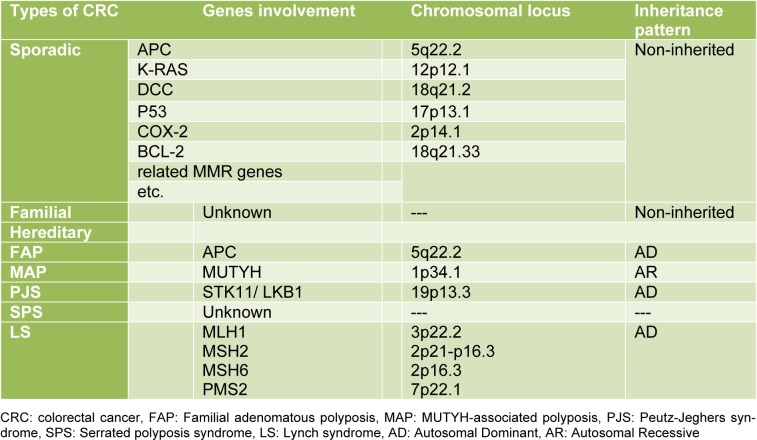
Types of colorectal cancer and their genes

**Table 2:**
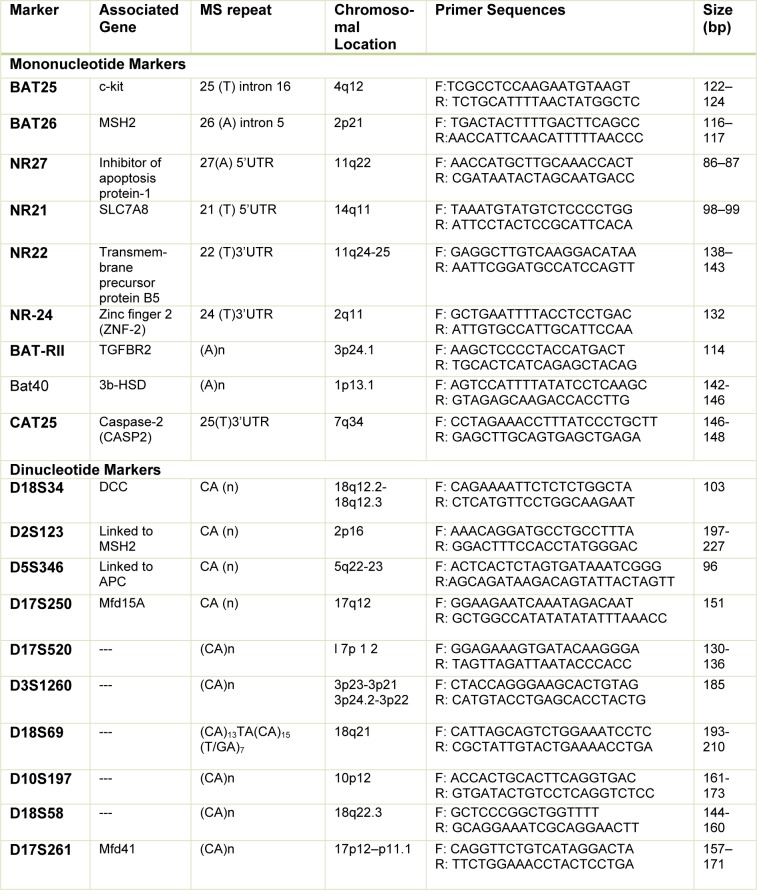
Microsatellite markers used to detect of MSI in CRC
